# Immune parameters to p67C antigen adjuvanted with ISA206VG correlate with protection against East Coast fever

**DOI:** 10.1016/j.vaccine.2018.01.087

**Published:** 2018-03-07

**Authors:** Anna Lacasta, Stephen Mwalimu, Elisabeth Kibwana, Rosemary Saya, Elias Awino, Thomas Njoroge, Jane Poole, Nicholas Ndiwa, Roger Pelle, Vishvanath Nene, Lucilla Steinaa

**Affiliations:** a Animal and Human Health (AHH), International Livestock Research Institute (ILRI), P.O. Box 30709, 00100 Nairobi, Kenya; b Research Methods Group, International Livestock Research Institute (ILRI), P.O. Box 30709, Nairobi, Kenya; c Biosciences eastern and central Africa-International Livestock Research Institute (BecA-ILRI) Hub, P.O. Box 30709, Nairobi, Kenya

**Keywords:** East Coast fever, Vaccinology, Subunit vaccine, Parasite, p67C antigen, *Theileria parva*

## Abstract

East Coast fever (ECF) is a lymphoproliferative disease caused by the tick-transmitted protozoan parasite *Theileria parva*. ECF is one of the most serious cattle tick-borne diseases in Sub-Saharan Africa. We have previously demonstrated that three doses of the C-terminal part of the sporozoite protein p67 (p67C) adjuvanted with ISA206VG confers partial protection against ECF at a herd level. We have tested the efficacy of two doses of this experimental vaccine, as reducing the vaccination regimen would facilitate its deployment in the field. We reconfirm that three antigen doses gave a significant level of protection to severe disease (46%, ECF score < 6) when compared with the control group, while two doses did not (23%). Animals receiving three doses of p67C developed higher antibody titers and CD4^+^ T-cell proliferation indices, than those which received two doses. A new panel of immune parameters were tested in order to identify factors correlating with protection: CD4^+^ proliferation index, total IgG, IgG1, IgG2 and IgM half maximal titers and neutralization capacity of the sera with and without complement. We show that some of the cellular and humoral immune responses provide preliminary correlates of protection.

## Highlights

• Three doses of p67C antigen generated stronger immune responses than two doses.
• Antibody titers and CD4^+^ T-cell proliferation correlated with protection against ECF.
• The number of doses could not be reduced from three to two without compromising the protection.

## 1 Introduction

East Coast fever (ECF) is a lethal disease of cattle caused by the tick transmitted protozoan parasite *Theileria parva*. The disease is a constraint for the development of the livestock industry in eastern, central and southern Africa because of the high mortality and morbidity caused by the disease. Cattle that recover from infection develop life-long immunity to re-challenge. This enabled the development of a live vaccine for ECF called “Infection and Treatment Method” (ITM), which is based on the simultaneous inoculation of a lethal dose of sporozoites and a long-acting oxytetracycline. This method of vaccination was used to create the “Muguga-cocktail” vaccine, which consists of three different *T. parva* sporozoite isolates and results in broad-spectrum immunity to ECF. This vaccine is now commercially available. However, the process of ITM has several limitations such as the need of a liquid nitrogen cold chain, use of antibiotics and animals can become carriers of the vaccine strains. Further, the vaccine is difficult to produce and therefore relatively expensive. This has led to attempts of finding alternative ways of inducing immunity to ECF (reviewed in [1], [2], [3], [4]).

It was previously demonstrated that MHC I-restricted CD8^+^ cytotoxic T-lymphocytes played a critical role in protection primed by ITM immunization [5], [6], but the humoral response also plays a role in mediating immunity. Sera from cattle immunized with *T. parva* Muguga (ITM) and repeatedly boosted by attaching adult infected ticks at intervals of two weeks, have high sporozoite-specific antibody titers capable of neutralizing infectivity of sporozoites *in vitro* [7], [8] and *in vivo* [9], the latter determined by mixing sporozoites and sera before injection into cattle. This led to a search for sporozoite proteins involved in the infection process, which could be used for induction of neutralizing antibodies and, hence, used for vaccination. Monoclonal antibodies with sporozoite neutralizing activity recognized a major surface coat protein in sporozoites [7], [10], called p67 due to its apparent molecular mass. The p67 protein is very conserved among cattle derived *T. parva* strains and consists of 709 amino acid residues, but it is polymorphic among buffalo derived stains [11], [12]. Thus, it is likely that p67 can function as a cross-protective immunogen for an anti-sporozoite vaccine, at least among cattle derived *T. parva* strains, and possibly for buffalo derived strains, as all p67 alleles share a high degree of sequence identity [12].

Several *in vivo* vaccine trial experiments have been performed with various constructs of p67 using full-length and fragments of recombinant protein with a range of adjuvants and gene based antigen delivery systems (reviewed in [1]). A consistent problem encountered in expressing full length p67 in *E. coli* in a soluble format has been protein instability, which affected yield and quality [13]. An 80 amino-acid (AA) peptide from the C-terminal part of p67 (p67C), containing epitopes recognized by sporozoite neutralizing monoclonal antibodies [14], can be expressed in stable manner and in high yield [13]. When tested using syringe challenge, immunization with p67C protein resulted in similar levels of protection to a nearly full-length version of the protein, p67_635_, 40 to 70% protection (ECF score < 6) against severe ECF relative to a control [13], [15], [16]. These results suggested that this fragment could replace near full-length protein. However, a general observation on the efficacy of immunization with p67 in field trials, where the challenge is achieved by infected ticks rather than a syringe challenge, was that protection levels relative to controls was lower, 20–30%, as approximately 50% of the animals in the control groups naturally recovered from tick challenge. The reason for this lower level of protection was not clear [15], [17], but it indicates that the efficacy of p67 under laboratory conditions needs to be improved.

Previous experiments had primarily established the efficacy of three doses of 450 μg of p67C given at four week intervals. However, the number of p67C antigen doses was not assessed. One of the objectives of this study was to test the possibility of reducing the number of doses from three to two, which would make the vaccination regimen more applicable for use in the field and lower the cost. Moreover, previous studies have not identified strong immune correlates of protection, which is an obstacle for vaccine development. Parameters, previously tested for correlation were: antibody titers (either total immunoglobulin (Ig) or IgG), neutralizing antibody titers and peripheral blood mononuclear cell (PBMC) proliferation indices. Only in one report [16], a correlation was found between the capability of sera to neutralize sporozoite infectivity and the level of protection. Hence, another objective of this study was to expand the panel of immune parameters to determine correlates with protection. This included CD4^+^ proliferation indices to p67C, total IgG, IgG1, IgG2 and IgM antibody half maximal titers, and a novel total antibody neutralizing assay, with and without complement, to help guide further improvements in optimizing the efficacy of p67C.

## 2 Material and methods

### 2.1 Experimental design of the *in vivo* experiment

Boran cattle (*Bos indicus*), 6 to 9 months old and negative for *T. parva* and *T. mutans* antibodies by ELISA [18] were used in the *in vivo* experiment. Thirty-two animals were randomly assigned into three experimental groups. In Group 1, ten animals were injected twice with 450 μg of purified p67C protein, and in Group 2, eleven animals were injected thrice with the same amount of protein. Antigen doses were administered subcutaneously 28 days apart. Group 1 animals were immunized for the first time at day 28 in the experiment, coinciding with the second dose of Group 2 animals. For Group 3, eleven animals were kept unvaccinated and used as a control group for challenge. The immunogen (p67C) was mixed with Montanide ISA206 VG adjuvant (Seppic) in a 1:1 ratio following the manufacturer’s instructions. The final volume injected in each animal was 2 ml. His-Tag p67C protein was expressed and purified as previously described [13].

Twenty-one days after the last boost, all animals were given a syringe challenge of 1 ml of *T. parva* Muguga sporozoites (stabilate #3087), diluted 1/100 in stabilate diluent (Minimum Essential Medium (MEM) Eagle (Gibco), 3.5% (w/v) bovine serum albumin (BSA, Sigma-Aldrich), 100 UI/ml penicillin (Sigma-Aldrich), 100 µg/ml streptomycin (Sigma-Aldrich) and 7.5% glycerol (v/v, Sigma-Aldrich). The stabilate, dilution and volume was identical to the ones used in earlier p67C experiments [11], [19], [20]. This dilution was reported to correspond to a lethal dose 70% (LD_70_), which implies that 70% of the control animals will be susceptible to the disease (ECF score ≥ 6). The challenge dose was administered in all animals sub-cutaneously over the parotid lymph node (local drainage lymph node). We did not include an adjuvant-alone control group because it is known that the use of Montanide ISA206 VG does not result in any background protection [15]. It is important to note that the sporozoite stabilate was titrated in an *in vivo* experiment before the current experiment, since the stabilate had not been used for 9 years. It was found that the stabilate had retained its potency.

After the challenge, all experimental cattle were monitored daily for changes in rectal temperatures and other clinical manifestations of ECF and the ECF scores were calculated (Rowland’s index) [21]. The index was used to define whether animals were susceptible (Index: 6–10, not protected) or immune (Index: 0–5.99, protected) to ECF, as previously described [15]. The humane end-point for the animals was determined by the institutional veterinarian based on clinical signs, such as: weakness, diarrhoea, staggering gait, dyspnoea and finally recumbency. The ECF scores included in the data analysis were from day 21 after the challenge. At this time point, the indices had stabilized and the experiment was terminated. Immune animals were monitored for an extra week in order to observe if there were further development in the indices. The animal experiment was approved by ILRI’s Institute Animal Care and Use Committee (IACUC number 2014.09). One-way ANOVA was used for comparison of the ECF scores between groups (Rowland’s Index), assumptions for normality were checked. The Fisher’s exact test was used for testing significance between groups of protected versus non-protected animals (binary 0/1).

### 2.2 ELISAs for detection of p67C specific antibody isotypes/subtypes

p67C specific antibodies in bovine sera were detected by means of an ELISA assay. p67C protein was coated at 0.5 µg/ml (in phosphate buffered saline, PBS) overnight at 4 °C on Maxisorp ELISA plates (Nunc). After blocking the plates for an hour with 0.2% casein (Sigma-Aldrich) and 0.1% Tween-20 (Merck) in PBS (blocking buffer), sera were added to the plate in 3-fold dilution series in duplicates starting from 1/100. The presence of the bovine antibodies was detected using the following antibodies, depending on the isotype/subtype of interest: sheep anti-bovine IgM:HRP, sheep anti-bovine IgG:HRP, sheep anti-bovine IgG1:HRP or sheep anti-bovine IgG2:HRP (all from AbD Serotec), all used at a dilution of 1:1,000 in blocking buffer. The reaction was developed using the following substrate buffer: 2,2′-azino-di-[3-ethyl-benzothoazolines-6-sulfonic acid] diammonium salt (ABTS, Sigma-Aldrich) at 134 µg/ml, 0.075% H_2_O_2_ (Sigma-Aldrich), 175 mM Na_2_HPO_4_ (Sigma-Aldrich) and 200 mM citric acid (Sigma-Aldrich), at pH 4. The reaction was incubated for 30 min at 37 °C in the dark and read at 405 nm in the Synergy HT ELISA reader (BioTek). Four washes were performed between every step using PBS Tween-20 (0.1%).

### 2.3 Calculation of half maximal antibody titers

Bovine sera were diluted in 3-fold dilution series, starting from 1/100 up to 1/72,900 using blocking buffer. This generated a logarithmic curve using the average of the optical density (OD) values of each serum dilution after subtracting the blank value. The principle followed was identical to the one used for calculation of the EC50 in a dose-response drug analysis. Half maximal antibody titers were identified using a nonlinear regression (curve fit) asymmetric (five parameters) using Graph Pad Prism version 7.0b for Mac (GraphPad software). A 2-sample Student’s *t*-test was used to compare animal groups. Due to small sample sizes a random permutation test was used to calculate the *t*-test probability, using random allocations of the data to generate a distribution for the t-statistic. This method was used for all comparisons except at day 28 after first immunization where all control animals had undetectable titers and hence a 1-sample *t*-test was used.

### 2.4 Sporozoite neutralizing assay based on anti-PIM cellular ELISA

Sera were tested for their ability to inhibit the *in vitro* infectivity of *Theileria parva* sporozoites. Sera were first complement inactivated at 56 °C for 30 min and then diluted 1/50 in Roswell Park Memorial Institute (RPMI) 1640 medium supplemented with either 1% rabbit serum or 5% heat inactivated rabbit serum (Cedarline). Sera were incubated for 10 min with 0.312 *T. parva* infected acini/well (approximately 13,000 sporozoites/well) of the #08/17 sporozoite batch diluted in RPMI 2% heat-inactivated fetal calf serum (FCS, Gibco) in a Corning 3799 plate at 37 °C in a CO_2_ incubator. The final volume per well was 100 μl (50 μl serum and 50 μl sporozoites, final sera dilution was 1/100). After the incubation, fresh Ficoll-isolated PBMC (5 × 10^5^ in 50 µl) in complete RPMI (cRPMI: RPMI 1640 culture medium (Sigma-Aldrich) containing 10% heat-inactivated FCS (Gibco), 2 mM l-glutamine (Sigma-Aldrich), 1 µg/ml gentamycin (Carl-Roth), 100 UI/ml penicillin (Sigma-Aldrich), 100 µg/ml streptomycin (Sigma-Aldrich) and 5 × 10^−5^ M 2-mercapthoethanol (BDH)) were added to the wells with the sera and sporozoites (150 µl final volume), and this was incubated for one hour at 37 °C in a CO_2_ incubator. The multiplicity of infection (MOI) was 0.026, which means 1 sporozoite every ∼40 cells and this was sufficient to turn 100% of the infection control wells positive for infection. Afterwards, the plates were centrifuged at 600*g* for 5 min, supernatants were removed, plates were gently vortexed and the cells were finally resuspended in 150 µl of cRPMI and incubated at 37 °C in a CO_2_ incubator for 14 days. The media was changed every 2–3 days and the infected cells were detected by means of an anti-PIM (*T. parva* polymorphic immunodominant molecule) cellular ELISA. Each serum at each dilution/supplementation was tested in ten different wells.

Infected and non-infected control PBMCs were permeabilized overnight at 4 °C with PBS 0.1% saponin (Merk), after fixing using 0.2% paraformaldehyde (diluted in PBS, Sigma-Aldrich) for 30 min at room temperature. Infected cells were detected using a monoclonal antibody specific for *T. parva* PIM protein (ILRI hybridoma clone ILS40.2) diluted at 0.5 µg/ml in diluent buffer (PBS 0.1% saponin and 10% FCS, Gibco) and the secondary antibody, HRP-conjugated goat anti-mouse antibody (A4416, Sigma-Aldrich), was diluted 1/5000 in diluent buffer. Primary antibody was incubated for 2 h at 37 °C, followed by two washes using PBS 0.1% saponin. The secondary antibody was incubated for one hour at 37 °C, and followed by 2 washes with PBS. The reaction was developed for 10 min using 60 µl of TMB plus 2 (Kem-En-Tec Diagnostics) and stopped using 60 µl of 0.5 M sulfuric acid (Sigma-Aldrich). 100 µl per well of the colored solution were transferred to a Maxisorp ELISA plate (Nunc). Reactions were read at 450 nm using the Synergy HT ELISA reader (BioTek). A well was considered positive for infection when the OD was more than double the value of the negative control (non-infected PBMCs). The results are shown as the percentage of neutralized wells, negative for infection.

### 2.5 CD4^+^ T-cell isolation with MACS beads and ^3^H-thymidine proliferation assay

CD4^+^ T-cells were purified as previously described [22]. CD4^+^ T-cells were stained with anti-bovine CD4 ascites (ILRI hybridoma clone ILA11), diluted 1/500. Purified CD4^+^ T-cells were used in a ^3^H-thymidine proliferation assay.

Cellular proliferation to p67C purified protein and p67C synthetic peptides (seven 25-mer overlapping peptides covering the full p67C sequence, Mimotopes Pty.) was analyzed by means of a ^3^H-thymidine incorporation assay. Briefly, purified CD4^+^ T-cells (2 × 10^5^/well) from the vaccinated animals were incubated for 4 days at 37 °C in a CO_2_ incubator with different stimuli in triplicates. The included stimuli were p67C protein at 100, 20, 4 and 0.8 µg/ml (equivalent to 12.6, 2.5, 0.5 and 0.1 µM, respectively) and a pool of the 25-mer p67C peptides, each at 2, 0.4, 0.08 and 0.016 µM. Media was used as a general negative control for the assay. Two extra negative controls were also included: *T. parva* Tp2 antigen peptide pool (Mimotopes Pty.) and ovalbumin (Sigma-Aldrich) as controls for p67C peptide pool and p67C protein, respectively, at the same concentrations. Concanavalin A at 2.5 µg/ml was used as a positive control. After 4 days of incubation, the cells were pulsed with 0.5 µCi/well of methyl-^3^H-thymidine (American Radiolabeled Chemicals). The plates were incubated at 37 °C in a CO_2_ incubator for a minimum of 8 h more and then harvested with a FilterMate Harvester (Perkin Elmer) onto glass fiber matt filters. Afterwards, filters were dried and placed in an Omni Filter cassette (Perkin Elmer), 30 µl of Microscint^TM^ (Perkin Elmer) was added and the plates were sealed with Topseal (Perkin Elmer). Finally, the plates were read using TopCount NXT reader (Perkin Elmer). The assay was performed at day 0, before immunization, and at day 70, two weeks after the last boost. The results are expressed as fold-change indexes when comparing the stimuli with the media averages (negative controls), which never had CPM values under 1022. A one-way ANOVA was performed on natural log transformed data, to satisfy normality assumptions, to test the statistically significant differences between the immunization groups.

## 3 Results

### 3.1 Animals receiving three antigen doses develop higher p67C-specific antibody titers with sporozoite neutralizing capacity than those receiving two doses

**Fig. 1:**
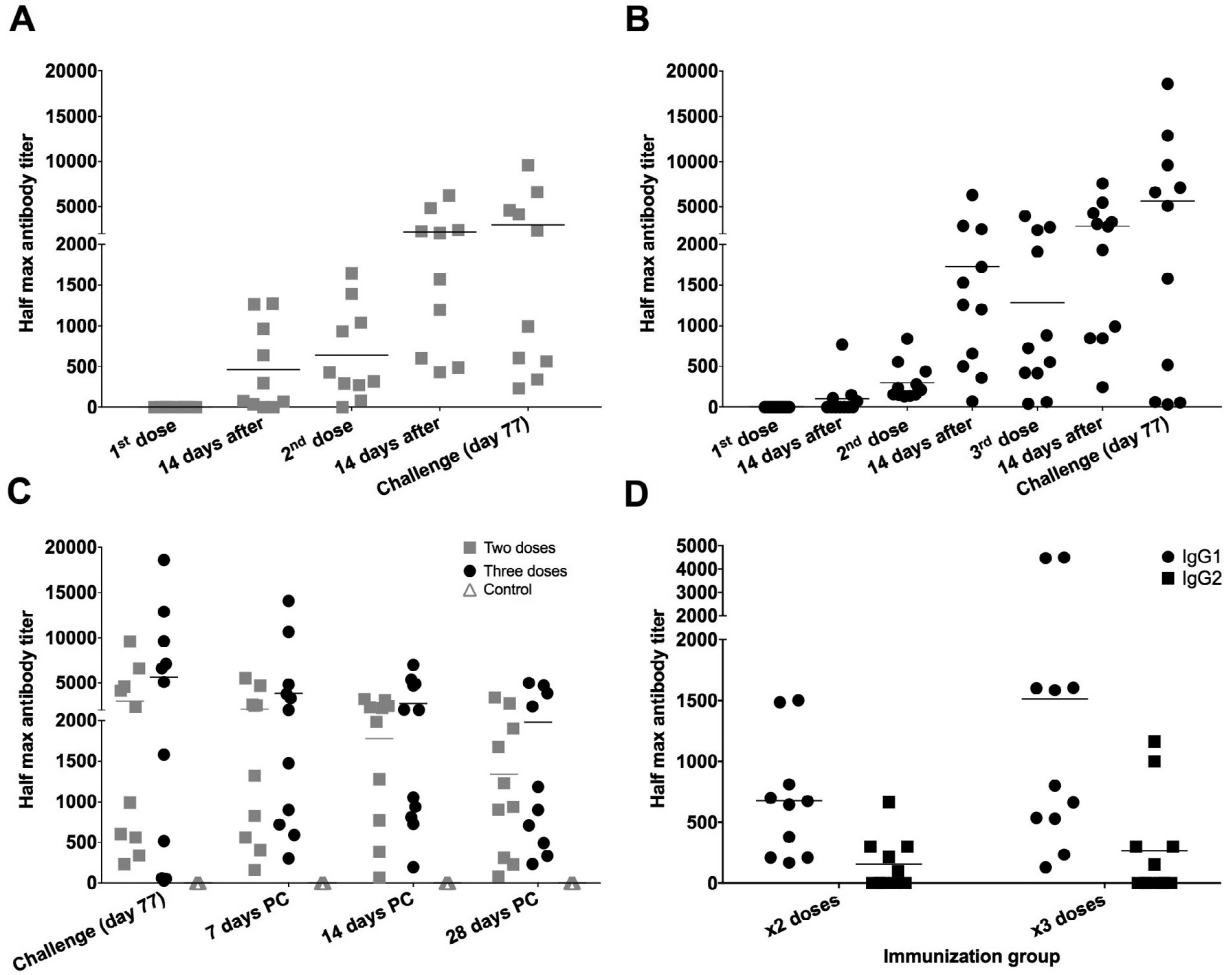
Kinetics of p67C specific IgG and IgG1 and IgG2 subtypes half max antibody titers. Antibody titers were measured by ELISA. (A) Group 1 IgG titers, two doses and (B) Group 2 IgG titers, three doses, before challenge; (C) comparative IgG antibody kinetics between Group 1 (2 doses) grey squares and Group 2 (3 doses) black dots, and the control group, grey triangles, post-challenge (PC). (D) p67C subtype specific antibody titers (IgG1 and IgG2) at the day of challenge for all immunized groups. Individual half maximal antibody titers are shown and the group means are also shown (black bar).

Group 1 and Group 2 animals were immunized two and three times with 450 μg of purified recombinant soluble p67C protein, respectively. A third group of animals (Group 3) was not immunized and was used as a challenge control group. Sera, collected every two weeks prior to challenge and every week post-challenge, were analyzed by ELISA for total IgG (Fig. 1A, B and C and supplementary table), IgG1, IgG2 (Fig. 1D and supplementary table) and IgM (data not shown).

Comparing the half maximal IgG antibody titers in the immunized groups to the control group, revealed significant differences appearing at day 28 after immunization (Student *t*-test, p = 0.006 for Group 1 and p = 0.001 for Group 2), and the significance was maintained for the rest of the experiment. It was evident that the level of total IgG antibody responses were boosted after each antigen dose (Fig. 1A and B) with antibody ranges from 234 to 9568 in Group 1 (IgG mean 3000; median 1670) and from 30 to 18598 in Group 2 (mean 5651; median 5096) at the day of challenge. Although, all animals had developed p67C-specific IgG antibodies at the day of challenge, the antibody titers were not increased post-challenge, which may reflect the low p67 antigen load presented by a single sporozoite challenge. Antibody titers started to wane about two weeks post-challenge and continued to drop until the last sampling time point, 28 days post-challenge. Control animals did not develop antibodies to p67C (Fig. 1C and supplementary table).

We also measured p67C specific IgG subtype responses at the day of challenge (Fig. 1C and D). The mean antibody titers were similar comparing Group 1 and Group 2 after the same number of doses, but it was higher for total IgG (Fig. 1C) and IgG1 and IgG2 subtypes (Fig. 1D) in Group 2 at the time of challenge (Group 1: mean IgG1 = 678 and IgG2 = 158; and Group 2 mean IgG1 = 1513 and IgG2 = 2656, Fig. 1D), although not significantly different. In both groups, the highest titers were found for the IgG1 isotype (Fig. 1D and supplementary table). No significant IgM titers were detected at any time point in any of the immunization groups.

**Fig. 2:**
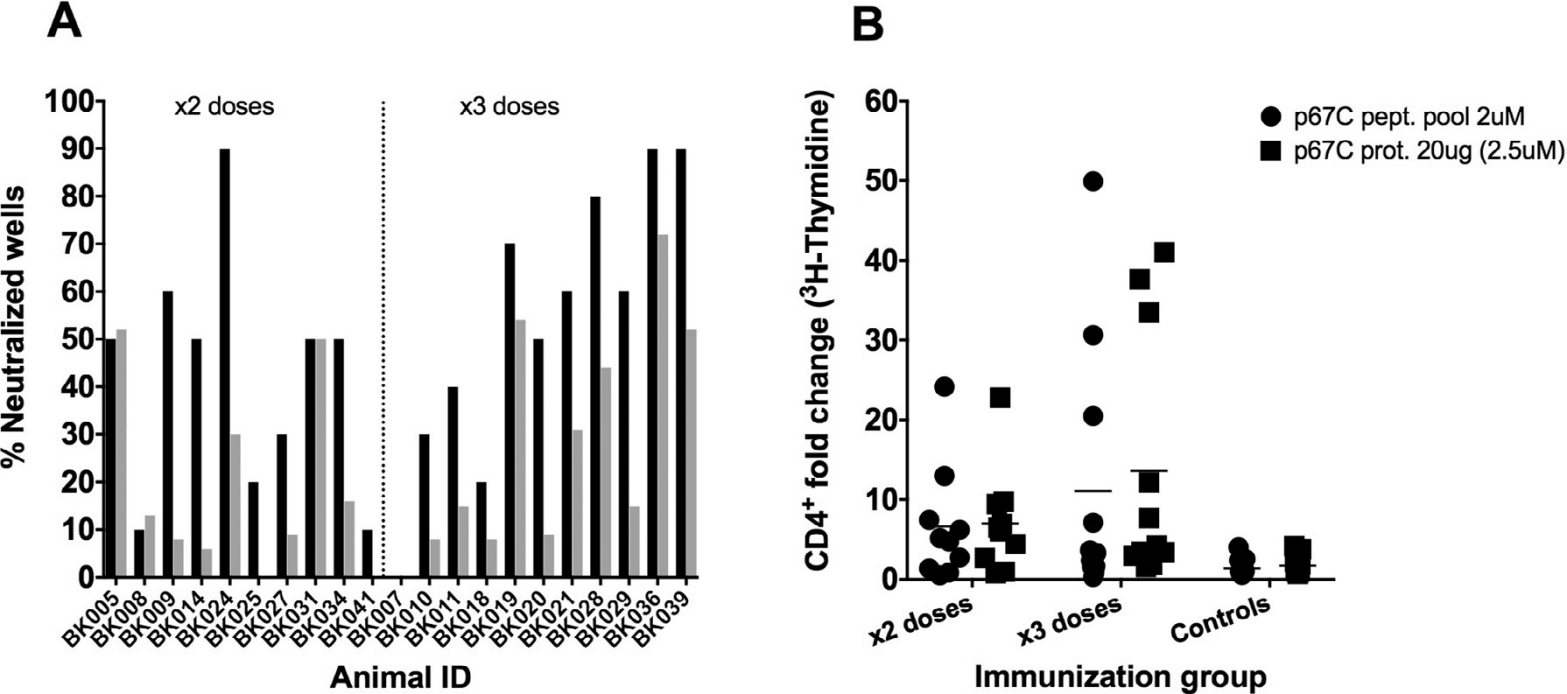
Individual antibody neutralizing capacity and p67C specific T-cell CD4^+^ responses. (A) Individual antibody neutralizing capacity of sera from Group 1 (2 doses) and 2 (3 doses) at day of challenge in the presence of 1% rabbit complement (black bars) or 5% heat inactivated rabbit complement (grey bars) and (B) p67C specific CD4^+^ T-cell proliferation index one week prior challenge (day 70) for all animal groups, measured by means of a ^3^H-thymidine based CD4^+^ proliferation assay. Two stimuli were used to measure the CD4^+^ proliferation: a pool of p67C overlapping 25-mer peptides at 2 μM/each and p67C protein at 20 μg/ml (equivalent to 2.5 μM). Animals are plotted individually and the average of each group of data is also present (black bar).

Sera from the time point of challenge were tested for neutralizing capacity by means of a sporozoite neutralization assay. Sera from immunized animals and two sera from the control group were analyzed as described in Materials and Methods. The sporozoite neutralizing capacity of most sera increased in the presence of complement (60% increase in some cases), reaching a level of 90% in some animals (Fig. 2A and supplementary table). In three cases, no effect of the presence of complement could be observed (BK005, BK008 and BK031). Complement seems to potentiate sporozoite neutralization, however, the neutralization capacity of the sera did not correlate with protection to ECF. On the other hand, the neutralization capacity of the antibodies correlates linearly with the IgG antibody titers (r = 0.49, p = 0.0236 for rabbit complement and r = 0.62, p = 0.0025 for heat-inactivated rabbit complement, supplementary figure A and B). There was no neutralizing capacity found in sera from day 0 (data not shown) or in sera from the control animals (supplementary table).

### 3.2 Strongest but variable CD4^+^ T-cell responses were developed after three doses of p67C antigen

To investigate the priming of T-helper cell responses to p67C, CD4^+^ T-cell proliferation assays were conducted at day 0 (pre-immunization) and two weeks after the last antigen boost (day 70) for all groups. MACS bead purified CD4^+^ T-cells were stimulated with soluble p67C protein or p67C overlapping synthetic peptides (Fig. 2B). As expected, no CD4^+^ responses were observed at day 0 with any stimuli in any of the groups (relatively to the controls, data not shown), nor in the control animals at day 70. Moreover, no CD4^+^ responses were observed to ovalbumin or a peptide pool from an unrelated antigen called Tp2, which were used as negative controls (data not shown).

In line with the antibody results, the animals injected three times with p67C developed higher p67C-specific CD4^+^ proliferative responses to the p67 protein (mean index = 13.6) than the ones receiving two doses mean index = 7; Fig. 2B and supplementary table). On a group level, both immunized groups had statistically significant CD4^+^ proliferative responses relative to the control group (ANOVA, p = 0.041 for p67C peptide pool, and p < 0.001 for p67C protein), but it was evident that there was a substantial variation in the proliferative indices between animals, and some showed indices equivalent to what was obtained for the control group animals. There were no significant differences in the proliferative response to p67C peptide pool and p67C protein between the two immunized groups.

For both immunized groups, there was a good correlation r = 0.7344 (p < 0.001) between the antibody titers and the CD4^+^ proliferation indices (p67C protein at 2.5 μM, supplementary figure C), with one outlier animal, BK036, which had a high antibody titer (half maximal titer = 9621) but a very low CD4^+^ index (using p67C protein 2.5 μM = 1.57). Excluding the mentioned animal, the correlation improves with an r equal to 0.8091 (p < 0.0001).

### 3.3 Three doses of p67C result in a significant level of protection compared to the control group

Three weeks after the last antigen boost, all animals were challenged with a 1/100 dilution of *T. parva* Muguga sporozoite stabilate 3087. The clinical outcome of parasite challenge was categorized according to the severity of disease reactions according to Rowland’s index [21], which uses 13 parameters for calculating a score between 0 and 10. Animals with a score below 6 were considered immune to ECF and animals with a score equal or above 6 were considered susceptible to disease.

Eight out of eleven animals from the control group were susceptible (ECF score ≥ 6) to ECF and three animals naturally recovered from the parasite challenge. Thus, the challenge behaved as expected, with 73% of the animals being susceptible to ECF, ∼LD_70_ (Table 1). However, one animal (BK017) was considered a non-reactor to challenge (ECF score of 0.68) as it had no parameters indicating infection, only a slight fever for a few days, but no schizonts nor piroplasms detected at any time point post-challenge. Moreover, it was not possible to detect parasite genomic DNA in whole blood at 14 days after infection, by means of *PIM* ORF PCR (data not shown). It is not clear why this animal was a non-reactor.

**Table 1:** Summary of ECF reactions in p67C-immunized and control cattle following challenge with *T. parva* Muguga sporozoites.

	×2 doses	×3 doses	Control
Immune (0–5.99)	5	8	3
Susceptible (6–10)	5	3	8
Protection (score ≤ 5.99)	50.0%	72.7%	27.3%

**Fig. 3:**
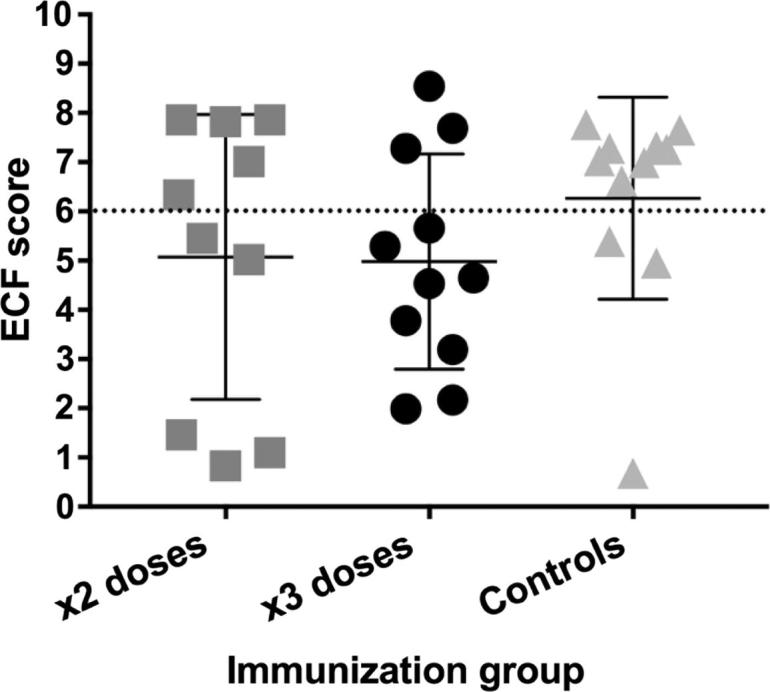
Immunity of cattle to ECF following sporozoite challenge. Animals are plotted individually and separated in groups: Group 1, two doses (grey squares), Group 2, three doses (black dots) and control animals (grey triangles). The mean of each group and the standard deviation is also shown. A cut off line separating immune (ECF score ≤ 5.99) and susceptible animals (ECF score ≥ 6) is shown as a black dotted line.

The proportion of susceptible animals in both immunized groups was lower, 5/10 in Group 1 and 3/11 in Group 2 versus 8/11 in the control group (Table 1). The proportion of immune animals was significantly higher in a 90% confidence in Group 2 compared to the control group (Fisher’s exact test, p = 0.086), but not for Group 1 (p = 0.387). In agreement with this, the mean ECF index scores for animals inoculated either two (p = 0.263) or three times (p = 0.216) with 450 μg of purified p67C protein were lower than the control group (Fig. 3). Thus, the level of protection achieved for each immunized group, after subtracting the ∼27% for the naturally recovered animals, due to the LD_70_ challenge, was 23% for Group 1 and 46% for Group 2.

### 3.4 Correlation of immune parameters to p67C with protection (ECF score)

**Fig. 4:**
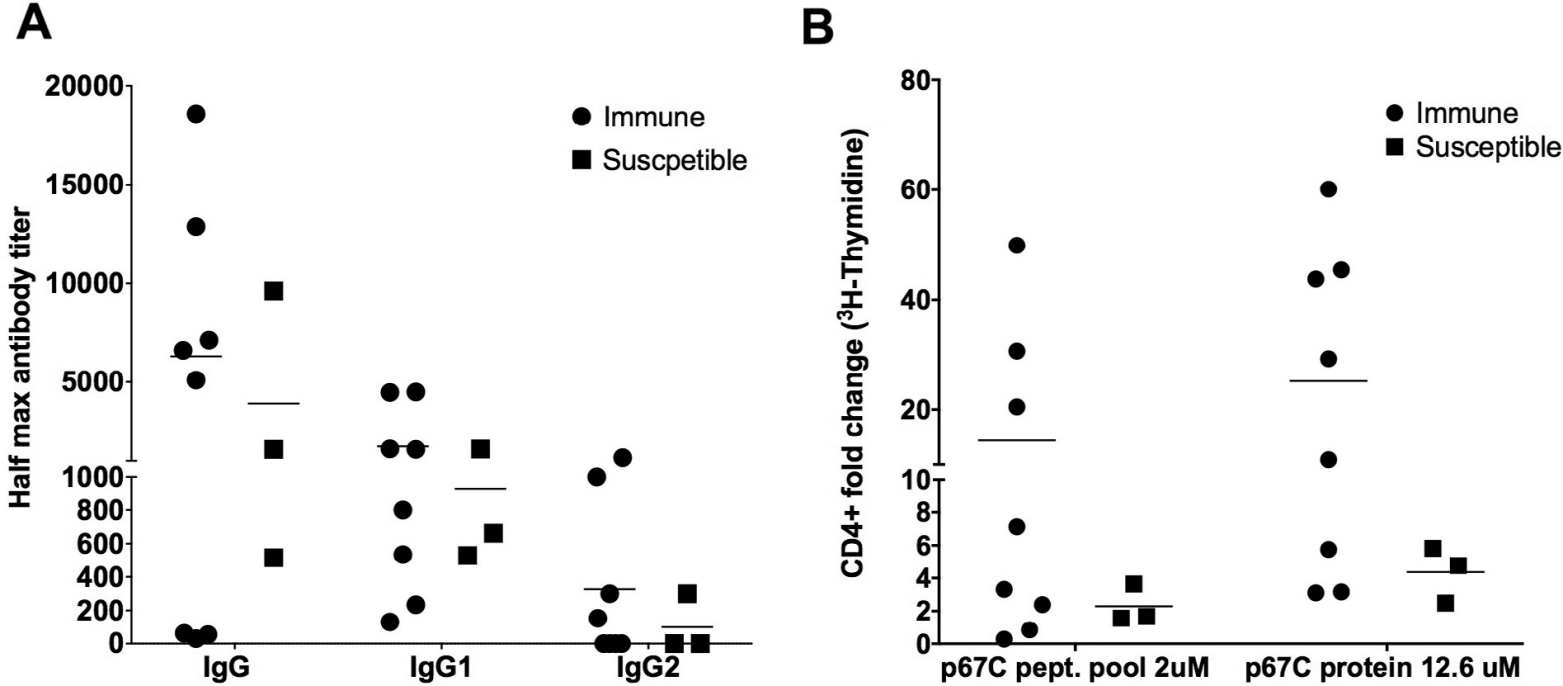
p67C specific immune responses in immune versus susceptible animals (Group 2). Group 2 (three doses) is parted in two, based on the ECF score: immune (ECF score ≤ 5.99) and susceptible (ECF score ≥ 6) for all the parameters. Animals are plotted individually and the average of each group is shown. (A) Subtype specific p67C antibody titers (IgG, IgG1 and IgG2) at the day of challenge (day 77), measured by ELISA and (B) p67C specific CD4^+^ T-cell proliferation index, one week prior challenge (day 70), measured by means of a ^3^H-thymidine based CD4^+^ proliferation assay in immune versus susceptible animals.

We assessed the measured immune parameters to determine if there were any correlates with protection to ECF. Results were analyzed for differences in the immune parameters among immune and susceptible animals. It was observed that the mean of the p67C specific antibody titers (IgG, IgG1 and IgG2, Fig. 4A) and the p67C specific CD4^+^ proliferation indices (p67C peptide pool and p67C protein, Fig. 4B) were all higher on average in the immune animals compared to the susceptible animals in Group 2 (three doses), indicating that these parameters indeed correlate with immunity and protection.

In addition to this analysis, a pairwise correlation was performed between immune parameters and the ECF score in four different combinations (Table 2): all immunized animals together (Group 1 and Group 2, data not shown), Group 1 (two doses, data not shown), Group 2 (three doses) and Group 2 without the outlier animal that had a high antibody titer but low CD4^+^ proliferation index (BK036).

**Table 2:** Correlation of immune parameters with protection (ECF score).

	×3 doses		×3 doses. minus BK036	
	Correlation (r)	p-value	Correlation (r)	p-value
CD4^+^ (p67C-prot.)	−0.627	**0.039**	−0.596	**0.069**
IgG	−0.487	0.128	−0.734	**0.016**
IgG1	−0.499	0.118	−0.606	**0.063**
IgG2	−0.640	**0.035**	−0.640	**0.047**

Significance ≥ 10% (p-value ≤ 0.100) in bold.

When analyzing data from Group 2, a negative correlation with the ECF score was found for all parameters, IgG, IgG1 and IgG2 titers at the day of challenge and CD4^+^ proliferative response at day 70, one week before challenge. Two were statistically significant: the CD4^+^ p67C-specific proliferation index (r = −0.627, p < 0.05) and the p67C specific IgG2 antibody isotype titers (r = −0.640, p < 0.05). As previously pointed out, there was an outlier animal (BK036) with high antibody titers but low CD4^+^ proliferation index, which did not fit into the pattern of the other immunized animals. There could be many unknown factors making this animal an atypical reactor. For this reason, we decided to also perform the analysis without this animal. In this case, all parameters correlated negatively with the ECF score with improved correlation coefficient values (r) and all parameters were statistically significant either at the 95% level or at the 90% level of confidence (Table 2). For Group 1 (two doses), none of the parameters correlated significantly with the ECF index (data not shown). This simply may be due to too few immune animals and generalized lower levels of antibody titers and CD4^+^ responses. It also remains a possibility that the correlation is non-linear at these lower levels. The linear correlation factor (r) was diluted when correlating all animals (two and three doses) together and hence also the significance, but IgG1 and IgG2 titers were still correlating at the 90% confidence level.

## 4 Discussion

Results from previous evaluations of p67 as a vaccine antigen using the full-length, nearly full-length version (p67_635_) and p67C, an 80 amino acid C-terminal portion of the protein, when mixed with adjuvant gave significant and similar levels of immunity to ECF (protection of 50–70%), using needle challenge under laboratory conditions. However, p67C had several advantages over the full-length protein when expressed in *E. coli*, such as better stability and higher expression yields [13], [15]. A very recent study has demonstrated stable expression of p67 without the transmembrane domain in mammalian cells [23]. It will be interesting to determine the efficacy of this antigen in vaccine trials.

It is remarkable that p67C (8 kDa), considering its small size, provides a high level of protection, suggesting it represents a critical target. Prior antigen dose experiments indicated that three doses of purified recombinant p67 or p67C protect against ECF [13], [15]. One of the objectives in the present study was to test if two doses of p67C would protect against ECF, as this regimen is more practical in the field. Our results show that a regimen of three doses, but not two, provides a statistically significant level of immunity to ECF (90% confidence).

The average levels of all tested immune parameters were higher in Group 2 (three doses) than in Group 1 (two doses). At the day of challenge, the half maximal IgG antibody titers were higher in Group 2 and so were the titers for the subtypes (IgG1 and IgG2). The CD4^+^ proliferation indices using both p67C protein and p67C overlapping peptides as stimuli were also higher in Group 2 than in Group 1. This strongly indicates that three doses of soluble p67C mixed with ISA206VG gives superior immune responses than two doses. This is corroborated by the results from the challenge, where Group 2 had a higher and statistically significant level of protection, with a ∼ 46% reduction of severe reactors (protection). Use of fewer doses of soluble p67C (GP64:p67C fusion protein) has previously only been described in one study [16], where two doses of insect cell extracts were administered, with an estimated p67C content of 10 μg/dose. The level of protection (36%) is between the results obtained for Group 1 (two doses) and Group 2 (three doses), corroborating the need of three doses of protein to achieve higher levels of protection. However, the dose of p67C protein was also reduced extensively in the mentioned study [16], but with a surprisingly high level of protection obtained, considering the dose of the antigen. Moreover, there is also evidence that too much antigen can lower the overall immune response [24], [25], so it remains a possibility that a smaller antigen dose could be beneficial – a possibility that we are currently testing. Increasing the number of doses or the quantity of the antigen (5 times x 1 mg) did not increase the protection when using full-length p67 [11], [19], [20], which makes it unlikely to be the case with p67C. Furthermore, three doses are far from an ideal vaccination regimen for a livestock vaccine and increasing the number of doses would make it even less feasible for a future field application. Thus, we did not contemplate the possibility of increasing the number of doses or the amount of protein per dose, even though this has never been tested before using p67C.

In the present study, animals with higher levels of antibody titers and CD4^+^ proliferation indices had increased chances of surviving the LD_70_ challenge. This is the first time that antibody titers and CD4^+^ T-cell responses have been found to correlate with protection (ECF score). Purified CD4^+^ cells were not used for proliferation experiments in previous p67C experiments [20], [26], [27], instead PBMC were used. In general, weak and inconsistent T-cell proliferation responses were previously observed using p67 full-length or nearly full-length (p67_635_). Different explanations were given for the lack of T-cell responses. In one report [20] the authors suggested that most of the responses were directed to the NS1 protein from *influenza* virus that was fused to the p67 protein in order to make it more immunogenic and stable, but the proliferative responses using this protein (NS1) alone were inconsistent as well. One study suggested that macrophages in the PBMCs could suppress the proliferative response [27]. In all previous studies, PBMCs were used in the proliferation assays, which in our hands results in more variation and background compared to using purified CD4^+^ cells (data not shown). This could account for some of the previously reported inconsistencies. Other factors that can influence the outcome of challenge when working with outbred farm animals, is the intrinsic genetic variations and unknown pre-experimental clinical histories of the animals. Ballingal and collaborators already showed that the genetic factors influenced the outcome of challenge [28]. They found a strong relation between the expression of alleles of the major histocompatibility complex class II (MHC-II) *DRB3* and the reaction of p67 vaccinated animals to challenge. Such factors could be responsible for the variability in antibody titers and CD4^+^ proliferation observed in our study. The small size of p67C may limit the number of available epitopes and thereby limit the CD4^+^ T-cell responses and antibody responses for some MHC-II alleles. However, in previous experiments, using full length p67, some animals did not respond either [19], [20], [27], making it unlikely that the variability in titers is due to the size of p67C alone. Nevertheless, it would be interesting to correlate the presence and expression of various genes/alleles involved in immunity, among others the MHC-II, with the experimental immunity/susceptibility and immune parameters (antibody titers and CD4^+^ proliferation indices).

A correlation between p67-specific antibody titers and protection against ECF has not been described before. However, many hypotheses were proposed to explain the lack of correlation, but most concluded that the quantity of the antibodies was not as important as the quality. One of the explanations was that the conformation of the recombinant p67 full-length protein was different from the native p67 protein and it was suggested that the additional antigenic determinants presented on the recombinant molecules may influence the nature of the immune response [16], [19], [20]. In the case of p67C, we attribute the differences between our results and the previous ones to variations in the assays used [13]. First, the antigen used for coating the ELISA plates in previous experiments was the nearly full-length p67_635_ instead of p67C, the antigen used in the *in vivo* experiments. Secondly, we measured half maximal titers rather than end-point titers, which were expressed as the last dilution where a particular figure was obtained (for example an O.D. > 0.1). Since the curve for O.D. versus dilution is not linear but sigmoid, slight variations in the end-point titer assay can result in large differences in the defined titer because it is determined on a rather flat area of the curve. In addition to this, quite small numbers of animals were used (seven animals) in previous experiments [13] and these factors could explain the lack of correlation. With more animals (eleven) and half maximal titer determination, we found a negative correlation between the p67C-specific IgG antibody titers and the ECF score in Group 2 (r = −0.734, p < 0.05). An interesting observation was that the p67C-specific IgG2 antibody isotype titers showed a better correlation with protection than IgG1. The correlation was not only statistically significant for Group 2 (with and without the outlier animal BK036), it was also significant at the 90% level of confidence when Group 1 and 2 were combined. The importance of this finding has to be explored in depth in further analyses since little is known about the respective roles of the IgG subclasses and the different cattle FcγRs in the triggering of immune functions (reviewed in [29]). The lack of information in this area merits for more studies on functional differences of the bovine isotypes/subtypes. In other fields, this has led to the discovery of seven isotypes in equine [30] and in the malaria field, the IgG3 and IgG4 isotypes were found to be of high importance [31], [32]. Unfortunately, it was not possible to analyze titers of other possible bovine antibody isotypes/subtypes as there are no secondary antibodies to other subtypes on the market. There is evidence that bovine possess the IgG3 antibody subtype [33], but no functional studies have been performed with this subtype. It remains a priority for the bovine vaccinology area to develop secondary antibodies to such non-characterized antibody subtypes.

Another parameter of interest would be the affinity of the p67C-specific antibodies generated. It is known that with each boost of antigen, B-cells with higher affinity would be stimulated and would produce higher titers of antibodies and also antibodies with higher affinities [34].

Besides correlating the quantitative parameters such as the antibody titers and the CD4^+^ T-cell proliferation indices with protection, we also analyzed the sporozoite neutralizing capacity of the sera at the day of challenge. Although, the neutralization capacity of the sera and the antibody titers (IgG) correlated with each other in the presence and in the absence of active complement, there was no correlation between the neutralizing activity and protection (ECF score). This lack of correlation has previously been reported in several studies [11], [19], [20]. Only in one study by Kaba and collaborators, a correlation was found between the neutralizing activity of the sera and the level of protection [16], but their assay was notably different compared to our *in vitro* neutralization method. In this study, we observed that the presence of complement enhances the neutralizing activity of the antibodies (up to 60% increase) and this could therefore be an active mechanism *in vivo* for clearance of sporozoites, either by exerting its effect directly on the sporozoites or upon infection where residual p67 protein remains for some time in the plasma membrane of the infected cells. In three cases, there was no influence of complement on the neutralizing capacity. Since these animals had equivalent IgG1 and IgG2 titers, this may be related with differences in fine epitope specificities as this is known to affect the complement activation [35]. It is a possibility that there could be entirely different functions of the antibodies in play, e.g., in malaria, protection was found to correlate with the antibody-dependent respiratory burst [36], the antibody-dependent cellular inhibition (ADCI) [37] and the opsonization/phagocytosis capacity of the antibodies [38]. These relationships are something to consider in future studies for *T. parva.*

The lack of correlation between the immune parameters and the protection in Group 1 remains a concern. We suspect that the reason for the lack of correlation could be partly due to the fewer immune animals in this group and partly be attributed to data not fitting a linear correlation. The correlation may be closer to a sigmoidal curve where the various immune parameters need to reach certain levels in order to be translated into lower ECF scores and below that, protection will not be achieved. Group 1 could be in this area of the curve, but for Group 2 (3 doses), the animals may fall into the slope of the curve. Unfortunately, there is a need of more observations (animals) to be able to confirm a sigmoidal relationship. Many advanced technologies are today available for analyzing the epitope specificity of sera, which help identifying correlates using high density peptide-chip microarrays [39]. In this context, it will be interesting to compare the epitope specificity of sera from immune and non-immune animals.

We have confirmed that a regimen of three antigen doses primes significant levels of immunity to ECF, but, unfortunately, two antigen doses did not. Thus, one of our objectives to reduce the immunization regimen to a more field friendly format remains to be achieved. We observed a substantial variability in the antibody titers and CD4^+^ T-cell responses, suggesting that other delivery systems should be explored to enhance the immune responses and decrease the variability. One way to increase antibody titers is to present the antigen in a particulate format, which is more immunogenic than soluble protein [40]. Given the small size of p67C, this should be possible using, e.g., different nanoparticle antigen delivery systems. Adding p67 sequences that contain known sporozoite neutralizing epitopes that map outside p67C could be beneficial. In this report, we have come closer to establishing a correlate with immunity to ECF. These correlates will be further explored in future experiments, and may help guide development of a more robust vaccine based on p67.

## 5 Conclusions

The results presented herein show that a regimen of three doses (Group 2) are superior to two doses (Group 1) when using soluble p67C protein adjuvanted with Montanide ISA206VG. All immune parameters from Group 2 (three doses) were stronger compared to Group 1 (two doses), resulting in ∼ 46% protection using an LD_70_ challenge. In addition, we showed preliminary correlates of protection with CD4^+^ T-cell proliferation index and IgG, IgG1 and IgG2 titers, which will help direct future experiments.

## Conflict of interest statement

The authors declare that they have no conflict of interest.

## Author’s contribution

AL planned the experiments together with LS and they supervised all experiments. AL performed ELISAs, neutralization assays and analyzed all the data generated. SM, EK, RS and EA, performed CD4 proliferation assay, were involved in ELISA’s and neutralization assays. TN sourced animals, sampled animals and monitored parameters for the ECF index. JP and NN helped in the data analysis and performed the statistical analysis. RP provided the p67C protein. VN contributed intellectually to experimental outlines and helped with the manuscript. LS oversaw the experimental design/study and, together with AL, writing of the manuscript. All authors have read and approved the final manuscript.
