# Supplementary material for: Immune parameters to p67C antigen adjuvanted with ISA206VG correlate with protection against East Coast fever
Source: Vaccine. 2018 Mar 7;36(11):1389–97. doi: 10.1016/j.vaccine.2018.01.087 (PMC5835154; doi:10.1016/j.vaccine.2018.01.087)
Supplement: Supplementary Table [file mmc1.pdf]

Vaccination regimen	Animal ID	ECF score	IgG titers day 77	IgG1 titers day 77	IgG2 titers day 77	CD4⁺ index Protein 2.5 uM	CD4⁺ index Peptides 2 uM	% NW 5% RC	% NW 5% HI-RC
3 doses	BK007	4.53	55	131	0	1.82	2.39	0	0
	BK010	7.69	1,580	663	300	3.43	1.67	30	8
	BK011	3.78	7,118	800	300	12.17	20.48	40	15
	BK018	4.65	30.31	235	153	4.22	0.28	20	8
	BK019	1.99	18,598	4,494	1,162	33.46	30.65	70	54
	BK020	7.28	516	529	0	3.50	3.64	50	9
	BK021	3.19	5,096	4,466	0	7.68	7.13	60	31
	BK028	2.17	12,880	1,584	1,000	37.68	3.32	80	44
	BK029	5.29	62	535	0	2.94	0.86	60	15
	BK036	8.54	9,621	1,600	0	1.57	1.57	90	72
	BK039	5.66	6,601	1,604	0	41.05	49.91	90	52
2 doses	BK005	1.10	6,616	1,500	300	4.42	4.77	50	52
	BK008	7.86	4,592	645	665	7.00	7.47	10	13
	BK009	0.83	2,659	1,485	215	9.47	24.16	60	8
	BK014	7.01	9,568	700	300	9.74	12.99	50	6
	BK024	7.86	4,146	674	100	2.71	6.22	90	30
	BK025	7.82	562	168	0	6.47	1.37	20	0
	BK027	6.33	992	379	0	6.03	2.30	30	9
	BK031	5.02	603	810	0	0.75	2.73	50	50
	BK034	5.45	234	210	0	0.97	0.50	50	16
	BK041	1.46	342	210	0	22.80	0.87	10	0
Controls	BK006	7.31	0	0	0	3.80	4.02	N/A	N/A
	BK012	7.28	0	0	0	4.15	2.55	N/A	N/A
	BK013	7.03	0	0	0	0.92	0.62	N/A	N/A
	BK015	7.65	0	0	0	1.22	0.19	N/A	N/A
	BK016	0.68	0	0	0	1.13	0.79	0	0
	BK022	7.26	0	0	0	2.23	1.04	N/A	N/A
	BK026	6.99	0	0	0	0.91	2.39	N/A	N/A
	BK030	4.95	0	0	0	1.86	1.01	N/A	N/A
	BK033	6.62	0	0	0	1.10	0.79	N/A	N/A
	BK037	5.39	0	0	0	0.67	0.89	0	0
	BK038	7.76	0	0	0	0.97	0.55	N/A	N/A

NW: neutralized wells; RC: rabbit complement; HI: heat inactivated
