# Supplementary material for: Immune parameters to p67C antigen adjuvanted with ISA206VG correlate with protection against East Coast fever
Source: Vaccine. 2018 Mar 7;36(11):1389–97. doi: 10.1016/j.vaccine.2018.01.087 (PMC5835154; doi:10.1016/j.vaccine.2018.01.087)
Supplement: Supplementary Figure [file mmc2.pdf]

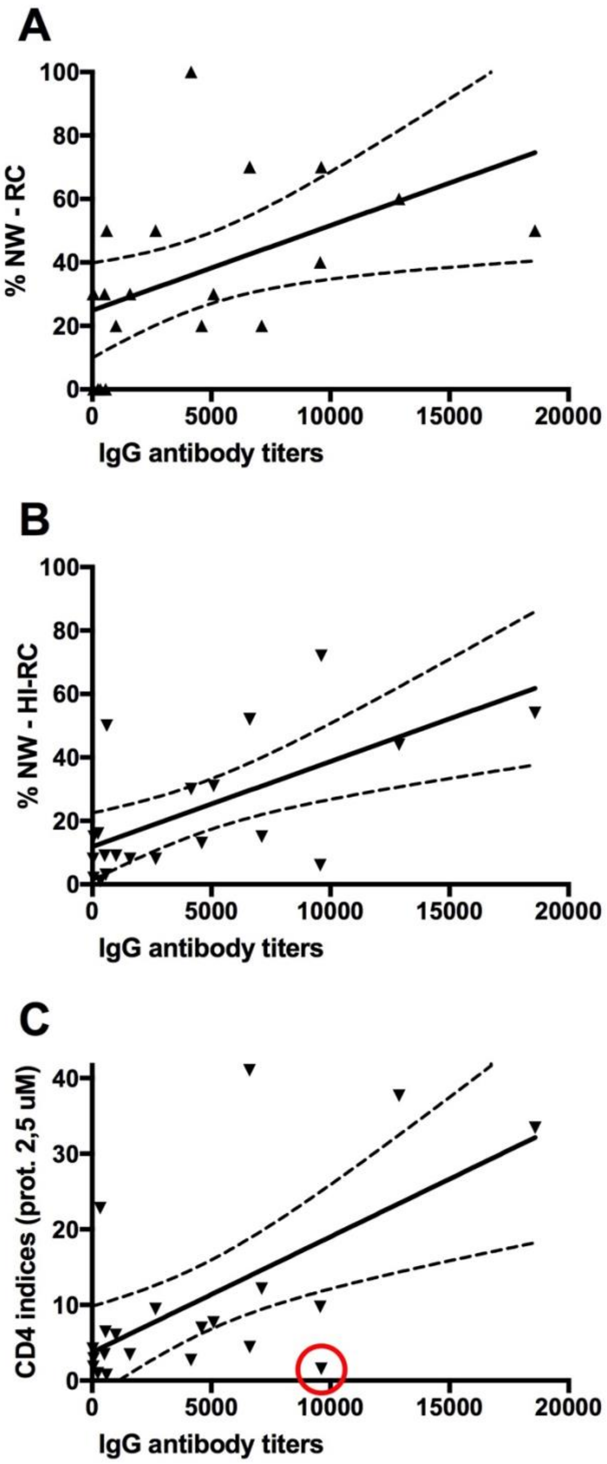

Supplementary figure. Correlation plot between antibody titers versus (A) Percentage of neutralized wells (NW) in the presence of 1% of rabbit complement (RC), r = 0.492 p-value = 0.0236; (B) percentage of NW in the presence of 5% of heat inactivated rabbit complement (HI-RC), r = 0.625 p-value = 0.002; and (C) CD4+ T-cell proliferation indices using 2.5 μM of p67C protein, r = 0.734 p-value < 0.001. BK036 (outlier) has been highlighted with a red circle, without this animal r = 0.809 p-value < 0.001. The line best fit is included in all plots as a black line and the 95% confidence around it is also shown with doted black lines.
